# Effects of Participating in a Volunteer Driving Program on Mental Health in Service Recipients and Volunteers

**DOI:** 10.1093/geroni/igab046.3130

**Published:** 2021-12-17

**Authors:** Machiko Tomita, Sutanuka Bhattacharjya

**Affiliations:** 1 University at Buffalo, State University of New York, Buffalo, New York, United States; 2 Georgia State University, Atlanta, Georgia, United States

## Abstract

Objectives: To assess the effects of a volunteer transportation program on mental health in riders and volunteers. Methods: A cross-sectional study (N=133, age ≥60) compared pre- and existing (≥2 years) riders, pre- and existing (≥2 years) volunteer drivers, and riders (Rs) and pre-volunteers (PreVs), representing general older adults. The cohorts belonged to a large, community volunteer organization. Outcome measures, depression and quality of life, were analyzed using ANCOVA. Percentages of people who wanted to go to specific destinations with available transportation were identified for pre-riders (PreRs) and Rs. Results: Rs had better depression scores (p<.001), no longer exhibited depressive symptoms (p=.005), and were better in quality of life (p=.002) than PreRs. Rs were similar to PreVs. PreRs’ major needs were going to medically related places (doctors’ offices – 74.4%, drug stores - 44.2%, hospitals – 37.2%) and basic living (grocery – 60.5%, clothing -37.2%). In Rs, these had significantly lowered, but still 40% wanted to go to doctors’ offices and 30%, grocery stores. Volunteer driver's (Vs)' depression (p=.009), health (p=.006), and social relationships (p=.004) were significantly better than PreVs'. Discussion: Although the use of free transportation up to four times a month may not be enough to improve perceived health for Rs, it was beneficial to prevent depression and increase quality of life. Since many PreVs were doing volunteer work other than driving, the type of volunteer work matters. Regularly helping people, in person, with their core needs for living resulted in positive outcomes for Vs.

